# Dr. Uwins' Case of Constipation

**Published:** 1813-12

**Authors:** David Uwins

**Affiliations:** Aylesbury, Bucks


### Medical and Physical Journal.

6 OF VOL. XXX.] DECEMBER, 1813. [NO. 178.

"For many fortunate discoveries in medicine, and for the detection of numerous errors, the world is indebted to the rapid circulation of Monthly Journals; and there never existed any work to which the faculty in EUROPE and AMERICA were under deeper obligations than to the Medical and Physical Journal of London, now forming a long, but an invaluable series."—RUSH.

### *To the Editor of the Medical and Physical Journal.*

SIR,

ON the 31st of last March, I was called to Quainton, a village about seven miles from this place, to attend Mr. King, a farmer, who was thought by the medical gentlemen already in attendance to be in a state of rapidly approaching dissolution. Mr. K. is a corpulent man, and has had for many years an umbilical rupture of such an extent as to include a very large portion of the intestinal canal. The first symptoms of his present complaint were a pain in the abdomen at some small distance from the orifice of protrusion, and sickness with obstinate costiveness: these symptoms had been present for some days before medical assistance was procured, and in the mean time purgatives of a common nature had been administered both by the mouth and the anus, without the effect of producing evacuation.

Before the medical gentleman who preceded me saw the patient, the pain which had been felt was in a very considerable degree mitigated, without the smallest abatement of the other symptoms; and the appearances at the time he was called in were such as to lead to the supposition of inflammation and strangulation having existed in some portion of the intestines, which were now followed by mortification. This opinion I also found every reason to adopt, and agreed with Mr. Hayward in the propriety of stating to the friends that the patient would probably not survive twenty-four hours. There was great prostration of the vital powers, pulse sinking and intermittent, incessant hiccup, and total freedom from pain.

The vomiting, it ought to have been stated, had every appearance of being *fœcal.* Under these circumstances, I recommended a more vigorous pursuit of the plan which had been already commenced—that of leaving the chance of cure entirely to the administration of opium. In addition to a grain of opium which had been ordered in the form of pill to be pretty frequently given, I directed two drachms of the tincture, with a pint of linseed decoction, to be injected in the way of enema every second or third hour. We left the patient in the evening, and by the report of next day I was delighted to learn that in a very short time after the injection of the opiate clyster, an evacuation of fæces took place, which promised and proved to be a signal of recovery. He from this time began to amend, and in a short period, without any further assistance from medicine, was left with no complaint but weakness.

Beside the satisfaction I felt in the recovery of my patient, I was pleased in the triumph of a principle which I am disposed to think is not in general sufficiently appreciated or acted upon, namely, the advantages promised by large and liberal doses of opium in circumstances of constipation of the bowel from spasmodic constriction. I care not by what name we designate this useful drug, antispasmodic, stimulant, or sedative; but I am anxious to impress on others my conviction that, notwithstanding its astringent qualities, there are cases (and the above is one of them) in which it is likely to prove almost the only *purgative* to be found in the materia medica, and that by a bold dash at the cause of the evil, disregarding minutiæ, we may frequently preserve and almost restore life.*

But to proceed with the account of my patient. On the 21st of last month I was again summoned to see him, and again found him much in the same state as above described. He had been without any evacuation downward exactly a week previously to my seeing him this last time, and during that period nine clysters had been administered, five common purgative ones, and four of the linseed decoction, with the same quantity of opium as before. The constipation now resisted even the large doses of laudanum, and I again almost abandoned every hope of his recovery. I, however, ordered one drachm of tobacco to be infused in a pint of water, and the infusion, with another two drachms of tincture of opium, to be injected as soon as possible. This last enema produced all the common effects of the tobacco thus

* I this day happened to open upon the chapter "*de Ileo*" in Dr. Heberden's commentaries, and found with much pleasure the following expressions respecting opium in this disease. After stating the several recommendatory qualities of the medicine in question, the author goes on to say, "Ea sententia multo potior mihi visa est quæ opium contra ileum prodesse statuit, quam quæ nocere."

given, given, without overcoming the stricture. I ought to have stated that in this last attack the evacuations by the mouth had still more decidedly the odor and color of fluid fæces, and that all the injections were retained. About the third or fourth day from the attack, something was discharged by the anus which the attendants confidently stated to be a worm, but which, when I saw it, had more the appearance of a portion of membranous substance, broken down and almost dissolved: it came away unaccompanied by fæces, and the constipation was not affected by its discharge.

Finding the effects of the tobacco clyster to be as above stated, I judged it prudent to discontinue its use, and ordered the following medicines to be continued:

℞. Mist. Assafœtid.

Aquæ font. āā. ℥iv.

Tinct. Opii, ʒij.

f. Enema Secundâ quâque horâ injiciend.

Sumat statim Hydrargyri, ℥iv.

Mitte Ol. Ricini, ℥ij. quo infricetur abdomen sæpe.

Four of the above injections were administered, and the quicksilver given the same night, after which the patient lay in a state almost of insensibility for about twenty-four hours, and the attendants were about to give him eight ounces more of quicksilver which I had ordered to be in readiness, when he unexpectedly expressed an inclination to stool, had a copious evacuation, and from that moment again gradually recovered. I did not see him again till yesterday, when I found him walking about his farm-yard. His bowels are now regular, but he is still very weak, and his legs have become œdematously swollen.

In reflecting upon the peculiarities of the above case, the following questions suggest themselves. What was the state of the intestines upon which depended the constipation? Was it an inverted action of the whole course of the canal; was it constriction from inflammation; or was it a case of *intus-ceptio*? And in regard to the remedies, we are led to inquire what share the opium had in the cure, and how much might be attributed to the operation of the crude mercury?

The pain which was felt in the first instance would lead to the supposition of local inflammatory action, while the retention of the clysters, and the fæcal vomiting, favor the conclusion of inverted action, which, by the way, when we reflect on the structure of the intestines, and especially the value of the colon, is not very conceivable. In regard to the share which the respective medicines had in the cure, I must repeat my conviction of the great benefit derived from

 the the opium, which, although less unequivocally and immediately operative in the second than in the first attack, still, in this last, I think, to say the least of it, preserved life, while the quicksilver or *vires naturæ* effected the restoration of due action in the bowels. It becomes a question how far the mercury operated at all, as there was no evacuation until the Thursday afternoon after it had been given on the Tuesday night.*

But I must conclude,—my intention was to publish facts, and not to propose theories; and I will no longer draw upon the pages of the Journal, or patience of its readers.

*Aylesbury, Bucks,* DAVID UWINS, M.D.

*Sept.* 15, 1813.

---

*To the Editor of the Medical and Physical Journal.*

* The attendants neglected to examine the fæces first discharged from the taking of the quicksilver.

